# A Rapid Review of Prescribing Education Interventions

**DOI:** 10.1007/s40670-020-01131-8

**Published:** 2020-11-16

**Authors:** Usmaan Omer, Evangelos Danopoulos, Martin Veysey, Paul Crampton, Gabrielle Finn

**Affiliations:** Health Professions Education Unit, Hull York Medical School, University of York, York, YO10 5DD UK

**Keywords:** Prescribing education, Medical students, Non-medical prescribers: curriculum design, WHO Guide to Good Prescribing

## Abstract

**Introduction:**

Many studies conducted on the causes and nature of prescribing errors have highlighted the inadequacy of teaching and training of prescribers. Subsequently, a rapid review was undertaken to update on the nature and effectiveness of educational interventions aimed at improving the prescribing skills and competencies.

**Methods:**

Twenty-two studies taking place between 2009 and 2019 were identified across nine databases.

**Results and Discussion:**

This review reinforced the importance of the WHO Guide to Good Prescribing to prescribing curriculum design as well as the effectiveness of small group teaching. However, it also highlighted the lack of innovation in prescribing education and lack of longitudinal follow-up regarding the effectiveness of prescribing education interventions.

## Introduction

Over time, deficiencies in prescribing education, such as a lack of practical prescribing training, a lack of linking theory to practice and the affordance of little attention towards generic prescribing skills, have led to the increasing emergence of prescribing errors [1]. A prescribing error is defined: “a clinically meaningful prescribing error occurring when... there is an unintentional significant reduction in the probability of treatment being timely and effective or increase in the risk of harm when compared with generally accepted practice” [2]. Errors in the prescription of medicines are currently one of the biggest dilemmas facing medicine and healthcare. Numerous studies have been conducted based upon prescribing errors and their impact on patient safety [3–5]. Adverse drug effects (ADEs) are found to be one of the main causes of injury to hospitalised patients [6], with over half of all prescribing errors considered as potentially harmful to patients, and 7.3% of these errors leading to life-threatening consequences [7].

Previously, only doctors and dentists held the legal authority to prescribe prescription-only medicines; however, this situation recently began to change globally, with either pharmacists or nurses or both obtaining the authority to prescribe independently [8]. The United Kingdom (UK) provides the most extensive rights to pharmacists and nurses, where doctors and dentists are known as medical prescribers (MPs) and other healthcare professionals who prescribe are known as non-medical prescribers (NMPs) [9]. The rationale of this development was to provide patients with quicker access to medicines. Not only would this decrease a very heavy workload within general practice but would also widen the use of the skills of pharmacists and nurses [10]. A small number of studies exploring the effectiveness of NMP prescribing have been encouraging, demonstrating that they are making clinically appropriate prescribing decisions [10, 11]. Baqir et al. found that pharmacist prescribers demonstrated an error rate of 0.3%; however, they advocate for further, larger scale research to be conducted on the prescribing practices of NMPs to obtain a clearer picture of the nature of errors NMPs can be prone to [12]. Cope et al. have also called for more research to investigate how NMPs are trained to prescribe safely and effectively [8].

Prescribing is overall a very complicated task requiring the amalgamation of knowledge of medicines, diagnostic and communication skills, an in-depth understanding of principles underpinning clinical pharmacology and an appreciation of risk and uncertainty [13]. Dornan et al. conducted research to determine the causes of prescription errors. They interviewed mainly recently graduated doctors and found that out of skill-based, rule-based and knowledge-based mistakes, rule-based mistakes were the main cause of prescribing errors. They reported that this suggests a lack in the ability of junior doctors to correctly apply the knowledge acquired in undergraduate education. This was supported by a consensus that students felt there was a lack of modules preparing them for the transition from theory to practice and current pharmacology education was not beneficial enough with regard to prescribing. It was concluded that rule-based mistakes were most likely to go unnoticed and inflict harm towards the patient [1].

Nazar et al. [14] built upon the research conducted by Dornan et al. [1] to delve further into the causes of prescribing errors. Their research implied that a lack of knowledge is not solely responsible for prescribing errors. They found that methods of teaching as well as the environment of prescribing also contribute toward prescribing errors. Audit Scotland questioned the adequacy of undergraduate medical education in preparing new doctors for rational and safe prescribing [15].

Previously, a systematic review was conducted by Kamarudin et al., examining previous work on educational interventions designed to enhance the prescribing competency of both medical and non-medical prescribers [16]. However, Kamarudin et al., as well as other systematic reviews on prescribing education interventions [17, 18], have only investigated the quantitatively measured effectiveness of interventions and omitted reviewing studies which qualitatively investigate the views and perspectives of students on the various interventions.

Given that previous literature reviews have omitted qualitative studies on prescribing education interventions, coupled with the advancement of the nature of educational interventions across the medical education continuum and the time elapsed since a previous review in this area, our aim was to perform a rapid systematic review to provide an update on the scope, nature and effectiveness of educational interventions aimed at developing the prescribing skills and competencies of medical and non-medical prescribers and investigate the views and perspectives of the students regarding different prescribing educational interventions.

## Methods

### Design

Given that previous literature reviews evaluating prescribing education interventions had been conducted, the aim was to investigate whether and to what extent the nature of these educational interventions had evolved in the last 10 years; therefore, a rapid review was deemed most appropriate. A rapid review is defined as a form of evidence synthesis that provides more timely information for decision-making as compared to a traditional systematic review. In addition, rapid reviews have been the preferred form of evidence synthesis for reviews aiming to serve as an update on previous reviews [19]. In addition, due to the heterogeneity of the studies and the inclusion of both qualitative and quantitative studies, the data was synthesised using a narrative approach [20].

### Search Strategy

The focus was towards identifying studies where an educational intervention was implemented in a curriculum to improve the prescribing skills of medical and/or non-medical prescribing students. Papers were screened from nine different databases, including MEDLINE, EMBASE, PsycINFO, Scopus, Academic Search Premier, CINAHL Complete, Cochrane Library, NIH PubMed and Google Scholar.

A search strategy was developed with the aid of a librarian from the University of York Library. The search terms entered into these databases were as follows:

**Table:** 

	Category	Keywords
AND	Prescribing	Prescribing OR Prescription* OR Prescriber*
AND	Education	Education OR Curriculum OR Training
AND	Intervention	Intervention* OR Innovation* OR Approach*
	Outcome	View* OR Perspective* OR Result* OR Effectiveness
AND	Population	Medical Student* OR Undergraduate OR Postgraduate OR Non-Medical Prescriber*

Search terms and strategy PROSPERO registration: CRD42019145576, Available from: https://www.crd.york.ac.uk/prospero/display_record.php?ID=CRD42019145576

### Study Selection

The inclusion criteria were if they were published in English, were full-text journal papers and evaluated an implemented educational intervention related to prescribing. Both qualitative and quantitative studies of any design taking place in medical schools and/or non-medical prescribing programmes were included, whether the intervention was evaluated through assessments or through qualitative student perspectives. However, they had to have taken place between the years 2009 and 2019. Papers were excluded if the educational interventions were not related to prescribing, and were systematic reviews, meeting reports, letters, opinion pieces or studies involving qualified doctors. The screening process took place in compliance with the PRISMA guidelines [21].

The titles and abstracts of the papers were reviewed by two authors to assess relevance of studies. Both authors held discussions regarding which papers should be included for full-text screening and an agreement was reached in a timely manner. Both authors also conducted full-text screening and, after agreeing upon 95% of the papers, selected them for data extraction.

### Data Extraction and Quality Appraisal

Initially, a small number of papers underwent dual data extraction by both Usmaan Omer and Evangelos Danopolous as recommended by Waffenschmidt et al. [22] based on study design, location, study aims, type and success of educational intervention, level of innovation and specific areas of prescribing targeted by intervention. The quality of each study was assessed using the Best Evidence Medical Education (BEME) scale [23]. As both authors agreed on the data extracted, data extraction of the remaining papers was conducted by UO and ED alone.

## Results

### Number of Studies

Overall, a total of 1137 papers were identified across all nine databases. Following the removal of duplicates, 696 papers remained, of which 634 were excluded for reasons including having no relevance to prescribing, studies not including medical and/or non-medical prescribing students as study cohorts or studies being conducted before 2009. After consultation between the two authors, it was agreed that 58 papers should be included for full-text screening. Following the process of full-text screening, 22 papers were included for the review. (PRISMA diagram included as Appendix)

### Study Characteristics

Of the 22 studies selected for the review, eight were randomised or non-randomised controlled trials, six before-and-after studies, five mixed-methods studies, two qualitative studies and one cross-sectional survey study (Tables 1, 2, 3, 4, and 5).

**Table 1:** Randomised controlled trials

Authors	Setting	Study design	Number of participants	Type of intervention	Learning outcome measures	Result of intervention	BEME score
Celebi et al., 2009 [45]	University of Tubingen, Germany	Randomised controlled trial	74 final year medical students; 36 early intervention (EI) group; 38 late intervention (LI) group	Intervention involved a week-long prescription training course including a seminar on ADRs and prescription errors, practical training based on a virtual case, prescription practice on wards, discussion sessions with lecturers on avoiding prescription errors. Intervention ended with assessment where student had to prescribe for two virtual cases	Could a DRP teaching module reduce prescription errors made by final year medical students in varying clinical contexts?	Students in the EI group committed significantly fewer prescribing errors after the intervention as compared to the LI group	5: Results are unequivocal
Kamat et al., 2012 [31]	Medical College and KEM Hospital, Mumbai, India	Randomised controlled trial	179 second-year medical students; 96 in intervention group; 83 in control group	Before intervention, themed lectures on specific topics delivered along with concept of P-drug and rational medicine use. After a pre-test, students randomised into 15 groups of 12, where 8 groups received case-based teaching (CBT). CBT involved discussing a case amongst a group and following the WHO 6 Steps. A month later, post-tests were administered for both intervention and control groups, where therapeutic problems similar to those encountered in the CBT were added	Could an intervention comprising CBT lead to more rational prescribing in students?	Students from the CBT groups attained higher marks than those from the control group and had more confidence to attempt more questions in the test	4: Results are clear and very likely to be true
Sikkens et al., 2018 [36]	VU University Medical Centre Amsterdam, Netherlands	Randomised controlled trial	356 fourth year medical students; 71 in intervention group; 281 in control group	e-Learning module offered to intervention group for 6 weeks. Module offered online through email and comprised 8 clinical scenarios based on the WHO GGP. Both intervention and control group completed pre- and post-tests	Can a problem-based, interactive e-learning module on antimicrobial prescribing improve antimicrobial prescribing skills and behaviours?	Students in the e-learning group scored significantly higher in both the post-test and OSCE simulation exercises as compared to control students. Students also expressed satisfaction for the e-learning course in a survey	5: Results are unequivocal
Thenrajan and Murugan 2016 [30]	Tertiary Care Medical College, Netherlands	Randomised controlled trial	50 Second Year Medical students; 25 in intervention group; 25 in control group	Two groups of medical students given introduction on prescription writing, prescribing format and WHO GGP for selecting preferred drug. Both groups taught prescription writing through five clinical conditions. Group 1 underwent patient-based teaching where they interacted with real patients, group 2 only trained in prescription writing. After 2 days, all students wrote prescriptions in standard format which were assessed	Is a patient-based teaching approach more effective in improving prescribing skills in medical students than case-based teaching?	Students who underwent patient-based learning performed much better than the control group who underwent case-based teaching. Students from test group provided high praise, stating the approach gave them higher motivation, focus, responsibility, empathy and helped with memory recall	5: Results are unequivocal
Tichelaar et al., 2016 [33]	VU University Medical Centre Amsterdam, Netherlands	Randomised controlled trial	251 second year medical students; 69 SMART group; 82 WHO group; 100 control group	Problem-based intervention using case studies. Students formed treatment plans either using the SMART goals, the WHO Guide to Good Prescribing or neither, which were assessed	Do SMART criteria improve treatment plans of medical students?	Treatment plans of students from SMART group had higher scores than the WHO and control groups, especially in treatment goal setting and monitoring	4: Results are clear and very likely to be true

**Table 2:** Non-randomised comparative control studies

Authors	Setting	Study design	Number of participants	Type of intervention	Learning outcome measures	Result of intervention	BEME score
Al Khaja et al., 2013 [34]	College of Medicine and Medical Sciences of the Arabian Gulf University, Bahrain	Non-randomised comparative control	910 first to third year medical students; 460 test group; 460 control group	2-h interactive session where students take 5–6 clinico-therapeutic case scenarios as carry-home exercises and these help in acquiring critical appraisal skills, use of drug formulary and prescribing skills. Prescriptions checked and formative feedback provided to students	Does attending an optional 2 h interactive prescribing session improve prescribing skill of attendees as opposed to non-attendees?	Attendees of the sessions performed significantly better in both exams and prescription writing skills than non-attendees	4: Results are clear and very likely to be true
Tayem et al., 2016 [39]	Arabian Gulf University, College of Medicine and Medical Sciences, Bahrain	Non-randomised comparative controlled study	108 second year medical students	Students attended a session which included discussing complete prescriptions for different cardiovascular diseases. Then students provided with role-play demonstrations on appropriate patient communication with patients regarding drug treatment. Role-play included correct way of relaying information to patient, such as explaining disease, aim of drug therapy and major ADRs.	Does a prescribing education intervention based on role-play demonstrations improve the prescribing communication skills of medical students?	Students felt that their skill in communicating prescriptions to patients had improved as had their confidence in prescription writing. They also felt that developing this skill would be more beneficial in small groups. Students who attended the session also performed better in the OSPE prescribing communication examination than controls in the three domains of introducing themselves to the patient, explaining the patient’s condition and providing instructions on drug use	5: Results are unequivocal

**Table 3:** Before-and-after studies

Authors	Setting	Study design	Number of participants	Type of intervention	Learning outcome measures	Result of intervention	BEME score
Gibson et al., 2014 [25]	University of Edinburgh, Scotland	Before-and-after study	183 final year medical students	196 junior doctor-led prescribing tutorials delivered to 183 final year medical students. Tutorials lasted 1 h, delivered throughout academic year and consisted of discussing clinical vignettes and agreeing on reaching principles of clinical management. Individual feedback given to students by tutor as well as group feedback. Tutorials ended with discussion about further patient management and prescribing principles	Could an intervention involving prescribing tutorials delivered by junior doctors improve the prescribing abilities and confidence of final year medical students?	Students reported increased confidence in their prescribing knowledge and skill as a result of attending tutorials and students who attended more tutorials performed better in the prescribing components of their final examinations	4: Results are clear and very likely to be true
Krishnaiah et al., 2013 [32]	Kempegowda Institute of Medical Sciences, Bangalore, India	Before-and-after study	78 second year medical students	Interactive teaching session using the WHO GGP, followed by hypothetical case studies	Does the WHO GGP improve the prescribing treatment plans of medical students?	WHO GGP-based teaching intervention lead to statistically significant improvement in treatment plans of medical students	4: Results are clear and very likely to be true
Newby et al., 2019 [24]	University of Newcastle, Australia	Before-and-after study	16 final year medical students	Clinical pharmacist-run tutorials on prescribing and drug calculations, including case-based scenarios	Does an 8-week pharmacist-led prescribing programme enhance the prescribing skills and confidence of final year medical students?	Students expressed significant improvement in generic prescribing confidence based on questionnaire results; however, they demonstrated small, non-significant improvements in prescribing appropriateness based on clinical scenario scores	5: Results are unequivocal
Paterson et al., 2015 [38]	University of Edinburgh and Edinburgh Napier Universities, Scotland	Before-and-after study	6 NMP students; 2 medical students	Intervention consisted of three cases commonly encountered in practice by foundation doctors and NMPs. Two scenarios required history-taking from simulated patient, suitable diagnosis and prescribing management plan. Third case was paper-based scenario and developed skills in medication review and recognising ADRs. Each scenario lasted 45 min, after which facilitator checked prescribing decision	Could a simulated inter-professional masterclass enhance inter-professional prescribing skills of medical and NMP students?	Readiness for Inter-professional Learning Scores (RIPLS) increased significantly from pre- to post-master class for both medical and NMP students as well as self-efficacy scores. In focus group discussions, participants expressed positive opinions of the master class. However, the cohort of participants was small and there would need to be further testing of the intervention	4: Results are clear and very likely to be true
Raghu et al., 2017 [28]	Tagore Medical College Chennai, India	Before-and-after study	117 second year medical students	Sessions on rational prescribing included group discussions on previous prescriptions written by students, pointing out mistakes. Students then rewrote prescriptions and these were assessed for quality using the WHO GGP	Can group discussion sessions on rational prescribing and improve prescription writing skills in second year medical students?	There was a significant improvement seen in the overall prescription writing skills of the students post-intervention	3: Conclusions can probably be based on the results (exact figures of improvement not reported)
Wilcock and Strivens, 2015 [43]	School of Medicine, University of Liverpool, UK	Before-and-after study	48 fourth year medical students	After a pre-test questionnaire, students devised into six groups of 6–10 whom each underwent a 40-min tutorial on a specific drug, the drug efficacy and effectiveness, a range of prescribing considerations and various prescribing scenarios where different prescribing judgements could be made. Later, students ran these tutorials among themselves. 8 weeks after last session, post-test administered	Can short pharmacology tutorials over an extended period of time improve the prescribing critical thinking skills of medical students	Interventions did not appear to lead to clear, sustained improvements in medical students’ critical thinking	3: Conclusions can probably be drawn from results

**Table 4:** Qualitative studies

Authors	Setting	Study design	Number of participants	Type of intervention	Learning outcome measures	Result of intervention	BEME score
Bowskill et al., 2014 [26]	University of Nottingham, UK	Qualitative study	63 non-medical prescribing students	Mentoring scheme paired NMP students with qualified NMP mentors who provided support around the integration of prescribing theory and practice. Data was collected through surveys and semi-structured interviews recording perceptions and experiences of mentoring scheme	Is a mentoring scheme collaborating NMP students with qualified NMP mentors seen as useful in integrating their theoretical learning with clinical practice?	Students found mentors helpful for moral support and implementing prescribing into practice, but expressed difficulties in contextualising course knowledge into practice given academic demands	5: Results are unequivocal and comprehensive
Cooke et al., 2017 [37]	Queen’s University Belfast, Ireland	Qualitative study	19–10 fourth year medical students; 9 third year pharmacy students	Simulation-based IPE activity on patient actors and small-group deliberations in specific intervals during sessions	Does a simulation-based IPE activity enhance professional development and perceptions of working collaboratively in students when prescribing	Students gained a much better understanding of the role of others in the prescribing process and how important working collaboratively is. Also learnt about the empathetic aspect of patient-prescriber communication. However, there needs to be an analysis using quantitative methods to truly determine the success of the intervention	4: Results are clear and very likely to be true
Dekker et al., 2015 [40]	VU University Medical Centre Amsterdam, Netherlands	Qualitative study	31 first, third and final year medical students	First, third and fifth year medical students prepared consultation plan consisting of history-taking, physical examination, additional investigations and treatment plan based on WHO GGP a week before proper consultation. 3rd year student performed consultation with 5th year, whereas 1st year student compiled the medical record. Follow-up consultation also occurred later for treatment monitoring, after which a feasibility questionnaire was administered to patients, students and supervisors	How feasible are student-run clinics on improving the pharmacotherapeutic skills of future doctors?	Patients, students and supervisors all expressed positive perceptions of the SRCs, with students finding that it enhanced their feeing of having responsibility and thinking about differential diagnosis. Patients also expressed satisfaction with care they received. Supervisors stated that intervention had added value for medical education. However, assessment through tests would be needed to truly evaluate effectiveness of intervention	4: Results are clear and very likely to be true
Hauser et al., 2017 [35]	Faculty of Medicine, University of Cologne, Germany	Qualitative study	12 third–fifth year medical students	Intervention combined both traditional and innovative methods. Students given paper case based on patient becoming non-adherent. Students define learning goals by end of first PBL session and return 2 days later for second PBL session having conducted research upon learning goals. This was followed by workshop where students and tutors developed medication conversation guide based on aspects of drug treatment and patient participation. Optional simulated talks conducted at end of course lasting 15 min each and observed by two tutors and videotaped. Students also filled in portfolios, including own strengths and weaknesses in prescribing communication	Could a newly implemented elective in the medical curriculum improve the physician-patient communication skills of medical students when coming to prescribing medications?	Participants expressed high levels of satisfaction with the elective; however, outcomes were measured merely through perceptions and not through tests	3: Conclusions can probably be drawn from results
James et al., 2016 [44]	College of Medicine and Medical Sciences of the Arabian Gulf University, Bahrain	Qualitative study	116 second year medical students	After exposure to case scenarios, students first devised into small groups of 13–15 where they discussed rational prescribing and general format of prescription and chart order and how to use the BNF. Then, there was discussion of clinical scenarios. Later in the year, large-group session with whole class conducted discussing the same things as the small group discussions. Questionnaires were administered after both small- and large-group discussions and there was also an additional focus group discussion	How well are small-and large-group prescription-writing sessions received by medical students?	Students perceived small group learning much better than large group learning given there is more chance to ask questions and improves communication skills, problem solving skills and teamwork and leadership. However, no attempt by study to assess effectiveness of intervention through tests	4: Results are clear and very likely to be true
Tittle et al., 2014 [27]	Barts and the London School of Medicine, UK	Qualitative study	1110 final year medical students	Weekly 2-h teaching sessions in hospital consisting of small-group tutorials, pharmacist ward rounds and shadowing of ward pharmacists	Does a pharmacist-taught prescribing course improve the prescribing confidence of final year medical students?	Students taking part in focus group interviews expressed that the course improved their prescribing confidence and were happy with the role of the pharmacist as a teacher. However, there was no quantification of this in the study, so success of intervention cannot be fully determined	3: Conclusions can probably be based on results

**Table 5:** Cohort studies

Authors	Setting	Study design	Number of participants	Type of intervention	Learning outcome measures	Result of intervention	BEME score
Achike et al., 2014 [41]	University of Hattiesburg, MS, USA	Cohort survey study	108–88 second year medical students; 20 fourth year nursing students	Interactive workshop class. 10 groups consisting of 10–12 medical students and 2–3 nursing students. Inter-Professional Learning (IPL) class consisted of lecture about rational prescribing steps, small-group discussions on choice of drug for particular drug scenario, group presentations on drug choices and prescriptions and finally feedback and Q&A. WHO GGP significantly involved throughout intervention	Is a rational prescribing IPE class well-received by medical and nursing students?	Overall student perception of intervention was very positive based on survey responses, especially regarding interaction with other healthcare professionals. However, there was no form of assessing the impact of intervention on prescribing skill	4: Results are clear and very likely to be true
Keijsers et al., 2015 [29]	Utrecht Medical School, Netherlands	Longitudinal cohort study	1652 medical students across the programme	WHO-6-Step incorporated into medical curriculum as part of integrated, longitudinal learning programme in pharmacology and pharmacotherapy. WHO-6-Step method used in large lectures, small-group tutorials and small-group practical sessions	Could the implementation of the WHO-6-Step across all stages of a medical curriculum increase prescribing knowledge and writing skills of medical students?	Both Bachelor and Master’s students significantly outscored controls in areas of basic and applied pharmacological knowledge and pharmacotherapy skills. Students receiving intervention in both Bachelor and Master’s phases expressed greater appreciation of education and more confident in clinical practice	4: Results are clear and very likely to be true
Zgheib et al., 2011 [42]	American University of Beirut, Lebanon	Prospective cohort study	109 fourth year medical students	Investigating the effects of teaching clinical pharmacology over 18 months to fourth year medical students using a team-based learning (TBL) approach on medical student satisfaction and performance	Does a TBL approach for teaching clinical pharmacology improve student satisfaction and performance?	Students performed much better on tests pertaining to prescription writing and formulary after the course and expressed high satisfaction with the TBL format. However, further studies needed to assess longevity of improved performance	4: Results are clear and very likely to be true

### Types of Educational Interventions

#### Teaching and Mentoring from Healthcare Professionals Other than Faculty Members

Four case-based educational interventions included teaching and mentoring from qualified healthcare professionals other than faculty lecturers [24–27]. Two studies followed a group learning format using case-based scenarios [24, 25], one study used experiential learning through observations of real-life prescribing situations [27] and one study implemented a mentoring scheme between learner and expert [26].

Newby et al.’s study [24] included pharmacist-led tutorials using common case scenarios seen by junior doctors, and, similarly, Gibson and colleagues used clinical case scenarios in tutorials led by junior doctors, but these were discussed in small groups of students, who devised a clinical management plan for the patient in the clinical scenario. Tittle et al.’s study [27] used small-group tutorials with students shadowing pharmacists in clinical practice, where topics such as prescribing for acute medical emergencies, taking patient drug histories, discharge prescriptions and therapeutic drug monitoring were covered. Bowskill et al. [26] implemented a mentoring scheme in the NMP programme at Nottingham, where students were allocated an alumnus of the programme who would act as their prescribing mentor, aiding them in effectively integrating prescribing skills learnt during the programme into their area of clinical expertise.

The studies used different methods to evaluate the outcomes of their studies. Newby et al. [24] employed mixed methods to evaluate the benefits of these sessions, where students undertook a prescribing exercise and a prescribing confidence questionnaire before and after the implementation of the intervention alongside focus groups where selected students discussed the benefits and potential drawbacks of the tutorials. Post-intervention scores were significantly higher, and both the focus groups’ and questionnaires’ data indicated that the tutorials had improved prescribing confidence in students. Gibson et al. [25] also used end-of-session questionnaires but observed student examination performance as indicators of success. The results of the questionnaires showed that most students rated the tutorials as ‘excellent’, greatly enhancing their prescribing confidence, knowledge and skills with the role of the junior doctor as the teacher being well received. Both Tittle et al. [27] and Bowskill et al. [26] evaluated outcomes qualitatively through focus groups, semi-structured interviews and surveys. Tittle et al.’s [27] focus group results demonstrated positive perceptions for the intervention; the role of the clinical pharmacist as the teacher and the positive effect of the intervention on their prescribing confidence were recorded. However, Bowskill et al. [26] found that although students praised the scheme for helping contextualisation of prescribing into their specific area of practice, they felt that adequate support was already provided from colleagues and tutors.

### Interventions Designed Using and Featuring the WHO Guide to Good Prescribing

Six case-based interventions were conducted through exposing students to treatment-setting standards from the World Health Organization (WHO) Guide to Good Prescribing (GGP) to varying [28–33]. Two studies used a combination of didactic lectures and subsequent prescription-writing for specific paper case scenarios [28, 31], two studies implemented an individualised instruction approach where students were provided with the WHO GGP to use individually for creating treatment plans [32, 33], one study used an experiential approach where students learned through observing real-life patients [30] and one study implemented the WHO GGP across an entire curriculum and in a variety of teaching formats [29].

Keisjers et al. [29] made extensive use of the WHO GGP through incorporating it into a whole medical curriculum, where all pharmacology and pharmacotherapy modules were modelled according to the learning goals of the WHO GGP, and the guide was heavily featured during whole-group lectures, small-group tutorials and practical sessions. Kamat et al. [31] themed prior lectures and case-based tutorials (CBT) involving treatment of varying conditions such as diabetes mellitus, peptic ulcers and constipation on the six steps of the WHO GGP. Raghu et al. [28] recruited 117 second-year medical students and asked them to compile prescriptions for three case scenarios. After delivering rational prescribing sessions and subsequently asking for the prescriptions to be re-written, they assessed and provided feedback to the students according to the WHO GGP standards. Both Krishnaiah et al. and Tichelaar et al. [32, 33] required students to use the WHO GGP as an aid in compiling treatment plans for hypothetical case scenarios; however, the purpose of Tichelaar et al.’s study [33] was to compare the impact of the WHO GGP to the ‘SMART’ criteria of goal setting on treatment planning. Thenrajan et al.’s study [30] used a test and a control group, both of whom were exposed to the WHO GGP guidelines of selecting the preferred drug following a clinical diagnosis. After receiving five clinical scenarios, the test group underwent patient-based teaching where they would see real patients suffering from the same conditions seen in the clinical scenarios, whereas the control group underwent further prescription-writing training.

Outcomes by most studies were assessed through scoring the treatment plans and prescriptions written by students following the intervention. Both Raghu et al. and Krishnaiah et al. [28, 32] found student treatment plans to score higher post-intervention and compared to control groups. However, Tichelaar et al. [33] found the treatment plans of students using the SMART criteria to score higher than those who used the WHO GGP. Keisjers et al. [29] examined the impact of their curricular intervention through a formative standardised assessment testing basic pharmacological knowledge (testing factual knowledge), applied pharmacological knowledge (solving clinical scenarios) and pharmacotherapy skills as well as prescription-writing. The results demonstrated that both fourth- and sixth-year students receiving the WHO GGP intervention significantly outscored their control group peers.

### Self-Directed and Online Learning

Three studies involved interventions which included a component of self-directed or online learning [34–36]. Two studies incorporated their self-directed components of the intervention alongside PBL-based tutorials involving case-based scenarios [34, 35] and one study implemented an entirely individualised e-learning prescribing module [36].

Al Khaja and Sequiera [34] investigated the impact of an optional 2-h interactive prescribing skills session at the end of each pre-clerkship unit phase, where five to six clinical scenarios were discussed. Hauser et al. [35] required students enrolled in their study to collaborate with tutors to develop model patient–prescriber conversation guides. Following a PBL session on medication non-adherence where they defined learning goals, students conducted independent research on strategies to achieve their learning goals in anticipation of a second PBL session where they discussed results of their research findings, which was followed by the workshop where they devised their conversation guides. Sikkens et al. [36] designed a randomised controlled intervention where a group of fourth-year medical students were provided access to a 6-week e-learning module with eight clinical cases based on the WHO GGP.

The outcomes of these studies were assessed through observation of usual course assessment, where the scores of participants were higher as compared to those who had not been recruited for the study [34]; student reflections in the programme portfolio, where students expressed a high level of satisfaction with the intervention [35]; and through MCQ knowledge tests and OSCE simulations, where it was found that students exposed to the e-learning group performed significantly better and pass rates were much higher compared to the control group. Survey results also showed that students rated the e-learning module to have enhanced their prescribing confidence in antimicrobial therapy [36].

### Simulation and Role-play

Three studies implemented an educational intervention centred around learning through role-play and Simulation-Based Medical Education (SBME) [37–39]. Two studies implemented a mixed disciplinary small-group approach to their role-play method of teaching [37, 38] and one study used a large-group experimental observation approach [39].

Cooke et al. [37] split medical and pharmacy students into small mixed-disciplinary groups who consulted with simulated patients and subsequently devised a working diagnosis, a mock prescription and detailed management plan to explain to the simulated patient. Paterson et al. [38] collaborated medical and non-medical prescribing students into multidisciplinary groups where they would devise prescriptions for three scenarios, two with simulated patients and one paper based. Tayem et al.’s [39] large-group demonstration intervention used a student volunteer on patient communication with regard to drug treatment. The faculty member acted as the physician and the volunteer student acted as the patient. All students had opportunity to act as volunteers in these demonstrations.

Study outcomes were assessed through both qualitative and quantitative approaches. Cooke et al.’s [37] focus group participants expressed positive perceptions of the intervention in focus groups, stating the ability to apply theory into practice in a safe environment along with understanding the role of other healthcare professionals in prescribing. Paterson et al.’s [38] focus group discussions indicated that students positively received the master classes, praised the concept of working in small groups and gained a greater awareness and appreciation of the roles of other professionals in prescribing. They also used a pre- and post-readiness for inter-professional learning score (RIPLS) and self-efficacy score to evaluate the impact of the interprofessional simulation exercise. Tayem et al.’s [39] used recorded questionnaires, where students found the role-play demonstrations instructive, helping to enhance their ability to communicate drug therapy information effectively to patients, increase prescription-writing confidence and that they would like to be given further opportunities to undertake role-playing exercises in other facets of their medical education. Additionally, students attending focus groups reported that the educational intervention helped develop interaction skills with patients and that the exercise would be most effective within small groups. Moreover, OSCE scores of those attending these role-play sessions were higher than those of non-attendees.

### Peer-Based and Inter-Professional Learning

Two studies implemented educational interventions where either students from multiple stages of the medical programme were recruited for team-based learning or students from different degree programmes were brought together to partake in an inter-professional–based learning experience [40, 41]. One study implemented a small-group experiential learning approach under supervision [40] and one study used a blended approach of didactic lectures and case-based small-group learning [41].

Dekker et al. [40] recruited first-, third- and fifth-year medical students to take part in a pilot intervention involving student-run clinics (SRCs), where first-, third- and fifth-year medical students were tasked with collaborating in consultations with real patients with a supervisor overseeing the consultation. Like Dekker et al., Achike et al. [41] also conducted a pilot study. However, this intervention brought together both second-year medical and fourth-year nursing students for an inter-professional learning (IPL) class. The class consisted of a brief didactic lecture followed by a small-group discussion on a clinical scenario and group presentation. Outcomes were measured by Dekker et al. [40] through evaluation questionnaires by students, supervisors and patients, from which feedback was positive all-round, with the consensus that the SRC was safe, provided high level of care and was beneficial to the students [40]. Likewise, Achike et al. (2014) [41] administered feedback questionnaires to students before they left the class, which showed overall positive perceptions of the class, with students complementing interactions with students of other professions and learning more about the process of rational drug choice.

Two studies implemented peer-based learning between students of the same cohort [42, 43]. Both studies implemented small-group teaching; however, one of these also incorporated large-group discussions at the end of the session [42] and the other implemented specific tutorials on a single topic [43].

Zgheib et al.’s study [42] included six clinical pharmacology sessions which were delivered twice monthly over a period of 3 months, of which five were team-based learning (TBL) sessions including activities such as compiling of group prescriptions and group formularies, small-group work on MCQs eventually being joined into whole-class discussion on answers, group work on clinical scenarios and their appropriate prescribing decisions. Wilcock and Strivens [43] conducted a study where a certain segment of the overall prescribing education intervention involved teaching between peers. Groups of six to ten students received one 40-min tutorial every 2 weeks on the medications aspirin, tiotropium and simvastatin. During the 6 weeks of these tutorials, one student in each group was asked to voluntarily provide their own tutorial to their peers on a fourth medication of their choice while following the same tutorial format.

The interventions were evaluated through multiple approaches. Zgheib et al. [42] graded group prescriptions, formularies and answers to case scenarios compiled in the sessions and provided students with the opportunity to mention the strengths and weaknesses of the course through completing course evaluation forms. The scores of the group prescriptions, formularies and case scenarios improved after each session and students expressed satisfaction with the format of the sessions, mentioning that they helped with improving their group interaction skills. Wilcock and Strivens [43] administered post-tests to their students, who demonstrated struggles on the ethics of prescribing and, although enjoyed delivering tutorials to their peers, did not appear to display sustained improvements in their critical thinking [43].

### Other Studies

Two studies did not fit under any specific theme as their objectives were of a more general nature [44, 45]. One study investigated whether case-based teaching was more effective in small-group or large-group settings. Small groups were made up of 13 to 15 students each and the large-group session included the entire cohort. Both sessions concluded with the distribution of questionnaires to students regarding their perceptions of the session. Focus group discussions also took place where a small number of students were asked to express their views and perspectives on both the small-group and large-group approaches. The results of both questionnaires and focus groups indicated a strong preference by students for the small-group teaching sessions [44].

Celebi et al. [45] conducted a study investigating whether a module on drug-related problems (DRPs) could help reduce the number of prescribing errors. Group 1 underwent the week-long prescription training course followed by a week-long skills laboratory training period, while group 2 acted as the late intervention group by undergoing the week-long skills laboratory training before the prescription training course. Both groups underwent assessments before the training, a week later and at the end of the training programme. The training module included a 90-min seminar on adverse drug reactions (ADRs), prescribing errors and special needs patients. Another 90 min was dedicated to practical training based on a virtual case of congestive heart failure. The next 3 days involved the students practicing prescriptions for real-life patients every morning and discussing the real-life patient cases with lecturers in afternoon sessions, affording attention towards avoiding prescribing errors. At the end of the week, students were required to sit an examination with cases like assessment cases but with different diseases. The results of the assessments demonstrated a significant decrease in prescription errors. These results were more prominent in the early intervention group [45].

## Discussion

In the last 10 years, we found 22 studies which met the inclusion criteria of educational interventions aimed at improving the prescribing skills and competencies of medical and non-medical prescribing students. These showed that a considerable amount of studies continue to be conducted on the best educational approaches to improving prescribing skills; however, as reported by previous systematic reviews [17, 18], generalisability and validity continue to be limited due to the diversity and heterogeneity of the reported studies.

The most recent literature review on this topic was conducted by Kamarudin et al. [16], which reported that many interventions were designed based on the concepts of the WHO GGP. This review also found that prescribing education interventions continue to be designed using the main concepts of the WHO GGP, demonstrating that despite its publication being back in 1994, the guideline continues to be the leading model for safe and rational prescribing to this day. This assertion is aided by the positive results yielded by interventions designed around the WHO GGP, both in assessment and student perception [28–33].

Despite there being a range of different educational interventions to improve the teaching of prescribing, most of these interventions feature the heavy use of clinical case scenarios. Brauer et al. [46] report that clinical case scenarios are vital to problem-based learning in healthcare and to the development of clinical practice guidelines. This also applies to the WHO GGP, which consists of a plethora of case scenarios of various clinical areas such as diabetes, cancers and gastrointestinal, respiratory and cardiovascular disorders. Hence, the designing of effective prescribing educational interventions requires the inclusion of robust clinical scenarios as they can be applied to improving multiple aspects of prescribing competencies such as prescription-writing, prescribing communication and recognising of ADRs. In addition, apart from one study, all studies reported a high level of success regarding their interventions, whether through students attaining higher scores in traditional assessments, scored treatment plans and OSCEs in comparison to control groups or through students expressing positive views of the educational intervention.

Another theme to emerge from this review was the use of small-group teaching. Many of the interventions required multiple small groups of students to be created to deliver the teaching, with one study specifically evaluating the difference in effectiveness between small- and large-group teaching. Along with demonstrating high scores in assessments, small-group teaching was particularly perceived positively in qualitative interviews with students. NMP programmes consist of far less student numbers per cohort as compared to medical school programmes; however, studies introducing educational interventions to NMP programmes remain very low, as this review could only locate two studies involving NMP programmes, one introducing a mentoring scheme to NMP students and the other involving an IPL intervention with medical students. Given that certain areas of the literature indicate an incredibly low prescribing error rate of NMPs [12], the specific benefits of small-group teaching in the context of prescribing skill require further investigation.

Despite identifying a range of different educational interventions aimed at improving prescribing education, the level of innovation seen in these interventions appears to be low, given that most studies used orthodox teaching methods such as didactic lectures and group exercises throughout. In a literature review, Dearnley et al. [47] categorised innovation in medical education to include simulation; digital teaching aids; online/e-learning teaching and assessment; social media and virtual learning environments. Only three studies implemented a degree of innovation, where simulated and real-life patients and role-play were used. Here, although one of the studies failed to provide an insight into the content of the simulated consultations, when students were provided with the opportunity to use their prescribing skills on either simulated or real-life patients, their responses were overwhelmingly positive. Some of the studies mentioned the use of self-directed learning aided through an online e-learning system; however, it was unclear what content was included in these e-learning systems. None of the studies implemented the use of social media or innovative uses of virtual learning environments such as virtual reality with virtual patients. Most studies implemented interventions which, for the most part, were based on case scenarios on paper.

Although with the exception of one study, all interventions were reported to be successful in improving the prescribing skill and competency of students and were perceived positively, questions on their long-term effects upon prescribing practice of students beyond graduation and into their full-time clinical careers still remain as these studies failed to implement a longitudinal follow-up of whether their benefits on the prescribing practice of these students are sustained over a long period of time, as this would be a more reliable indicator of whether an educational intervention has achieved its desired outcome. Moreover, studies which only assessed the benefits of an intervention through the views and perspectives of the students undertaking them would be greatly enhanced if they utilised assessments and evaluated whether the scores of these assessments supported the positive viewpoints of the students.

Given that most studies only assess the short-term impact of educational interventions on prescribing practice, educators should also assess whether the positive impact of these interventions is sustained over a longer period as prescribers advance in their careers. Also, the WHO GGP continues to be a model from which prescribing educators design their teaching approaches. This could partly be due to it providing a comprehensive prescribing guidance on many areas of expertise using clinical case scenarios, something established as being core to problem-based learning. Given the lack of educational interventions being evaluated in NMP programmes, it would be prudent to design an intervention around the WHO GGP and evaluate its effectiveness in an NMP setting due to the existence of a variety of clinical areas of expertise in NMP programme cohorts.

This review did include certain limitations. As we limited the inclusion criteria to include studies involving students only, we could have included studies involving junior doctors. The search strategy also excluded non-English language papers. In addition, given that the papers we identified reported positive outcomes and perspectives as a result of the interventions, there is also the possibility of positive publication bias.

Overall, this review was able to retrieve a broad range of studies investigating various prescribing education interventions.

## Conclusion

Although a wide range of educational interventions to improve prescribing skills and competencies have been developed, despite their high success rate in the short term in both assessment and student perception, there still exists a lack of innovation in these interventions. Given that we are seeing other areas of medical education adapting their teaching approaches to be more innovative with the recent rise in technology, prescribing curricula also need to adapt and evaluate the scope of implementing educational approaches which utilise innovations such as virtual reality and explore areas where students can commit errors in a safe environment and learn from these to better their prescribing skills in preparation for real-life clinical practice.

## Figures and Tables

**Fig. 1:**
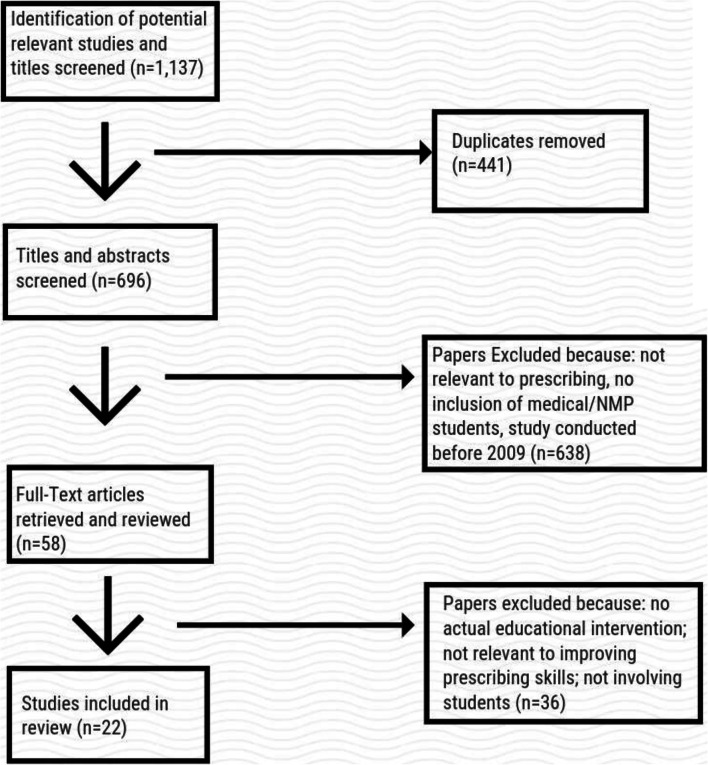
Flowchart of rapid review

## Data Availability

Not applicable
